# Tolerability of naso‐esophageal feeding tubes in dogs and cats at home: Retrospective review of 119 cases

**DOI:** 10.1111/jvim.16732

**Published:** 2023-10-25

**Authors:** Renaud Dumont, Julie Lemetayer, Loïc Desquilbet, Elodie Darnis

**Affiliations:** ^1^ Internal Medicine Department Veterinary Hospital Frégis Arcueil France; ^2^ Internal Medicine Department Veterinary Hospital Advetia Vélizy‐Villacoublay France; ^3^ Biostatistics and Clinical Epidemiology Department National Veterinay School of Alfort Maisons‐Alfort France

**Keywords:** anorexia, cats, dog, enteral feeding, nutritional support

## Abstract

**Background:**

The use of naso‐esophageal feeding tubes (NFT) at home could represent an alternative way to reduce the costs for owners and facilitate enteral feeding until recovery of a spontaneous appetite.

**Objective:**

To describe the use of NFT at home in dogs and cats and evaluate the satisfaction of owners and their capacity to handle the device.

**Animals:**

One hundred nineteen client‐owned animals (90 cats and 29 dogs) which remained anorexic during hospitalization and were discharged with NFT for at least 24 hours after placement.

**Methods:**

Medical records were reviewed retrospectively, and owners were contacted by telephone calls. Complications were reported according to their relative severity (minor and major). Owners were asked to report their experience and comfort with NFT management.

**Results:**

Naso‐esophageal feeding tubes were kept in place at home for a median of 6 days (range, 1‐17) and 62.2% (95% confidence interval [CI]: 53.3‐70.7) of animals recovered a spontaneous appetite while wearing NFT, 60% (95% CI: 44.4‐75.6) of the remaining animals recovered a spontaneous appetite after removal. Overall complication rate was 65.5% (95% CI: 57.0‐74.0), but only 18.5% (95% CI: 11.5‐25.5) required a consultation and no life‐threatening complication occurred. Owners were satisfied in 94.1% (95% CI: 89.9‐98.3) of cases.

**Conclusion and Clinical Importance:**

Although most animals discharged with NFT at home presented complications, no major adverse effects were reported and NFT were easily handled by owners. This study provides evidence that NFT can be well tolerated at home.

**Abbreviation**
NFT: naso‐esophageal feeding tube

## 1 INTRODUCTION

Inappetence in hospitalized dogs and cats is an important and recurrent complaint in clinical practice. Loss of appetite can result from several causes, including underlying pathological processes, stress, pain, medications, or dietary factors.^1^ Stress‐induced anorexia is hard to discriminate from other factors, often underestimated, and can adversely affect recovery from illness.^2^, ^3^ Therefore, recognition and management of stress in hospitalized cats and dogs is fundamental. Consensus guidelines on management of inappetent hospitalized cats have recently been published, highlighting strategies to reduce stress and encourage voluntary food intake.^4^ These measures include gentle human interactions, environment optimization, feeding considerations, and encouragement of positive experiences. Appetite stimulant drugs (eg, mirtazapine, capromorelin, cyproheptadine) represent interesting adjuvant strategies to promote appetite in hospitalized dogs and cats but can induce adverse effects and should be cautiously used to avoid overmedication and additional stress.^2^ Despite optimization of environment and feeding care, prolonged undernutrition persists in a number of animals, leading to several physiology alterations and life‐threatening complications.^5^ In addition, early enteral nutritional support has been associated with improved outcomes in several diseases,^6^ especially in pancreatitis,^7^, ^8^ parvoviral enteritis,^9^ septic peritonitis,^10^, ^11^ or protein‐losing enteropathy.^12^ Initiating early nutritional support by enteral route is preferred because it is the safest, most convenient, and best physiological way to provide nutritional support.^5^

Naso‐esophageal feeding tubes (NFT) represent a way to achieve early enteral nutrition and to deliver nutrients for animals who cannot ingest adequate calories on their own. Furthermore, feeding tubes can also be useful for dogs and cats with specific conditions for which intravenous fluid therapy can be deleterious and where sodium‐free enteral hydration could represent a less aggressive alternative.^13^ NFT are a relatively safe, noninvasive, and inexpensive device that can be placed or removed without general anesthesia, in systematically sick conscious animals or under mild sedation. Although indications for NFT placement are clear, their placement and maintenance can be frustrating. In fact, they are prone to obstruction because of the small diameter, require liquid diet exclusively, and might prevent voluntary food intake.^5^ In humans, some evidence recognizes the presence of naso‐esophageal or nasogastric tubes as a risk factor for aspiration pneumonia.^14^ Therefore, NFT are contraindicated in animals with protracted vomiting, esophageal diseases, poor gag reflex, or altered mental status.^1^, ^4^, ^5^ The use of NFT remains historically restricted to hospitalized dogs and cats based on clinician perception of poor tolerability and safety at home.^4^, ^5^ The use of NFT at home could represent an alternative way to reduce costs for the owners by shortening the duration of hospitalization and facilitates enteral feeding and liquid drugs administration until recovery of spontaneous appetite in animals for which esophageal tube placement under general anesthesia is unsafe.

According to the authors' knowledge, no study has properly evaluated the use of NFT at home in dogs and cats yet. In this context, the aims of the study were to describe the use of NFT at home in dogs and cats who remained anorexic during hospitalization, and to evaluate the satisfaction and capacity of owners to handle the device. Our hypothesis was that NFT can be used at home without major complications and easily handled by owners.

## 2 MATERIALS AND METHODS

### 2.1 Case selection and data collection

The medical records of hospitalized cats and dogs fed with NFT were retrospectively examined. The electronic files at the Centre Hospitalier Vétérinaire Frégis (Arcueil, France) were searched from January 2021 through June 2022 for the term “naso‐esophageal feeding tube.” A total of 553 files were examined for eligibility and cases were eligible for inclusion if (1) animals remained anorexic during hospitalization and (2) were discharged with NFT in place after a minimum in hospital observational period of 24 hours.

The following data were recorded both by electronic medical files examination and phone calls to owners: signalment, history, body weight, clinical data, duration of feeding tube use during hospitalization and at home, total duration of the feeding tube placement, reason for feeding tube removal, time from discharge to spontaneous return of appetite, difficulty with drug administration via feeding tube, need of more than 1 person to handle the device at home, and subjective global satisfaction of the owner was collected through a phone call, no more than 5 months after discharge. Owners were asked by phone to define the use of NFT as “simple,” “simple after some practice,” or “complex,” if the explanations of its use provided at the time of discharge were good enough, if they were alone to handle the tube or if 2 persons were needed, if they encountered difficulties to administrate medications through the tube (eg, mechanical problems, including difficulties to crush tablets into fine enough powder or NFT obstruction by medications), and if they were globally satisfied with the use of NFT at home. The consent of each owner for the use of the data collected was requested at the start of each phone call.

### 2.2 NFT placement and refeeding protocols

The placement of NFT was standardized. Enteral feeding tubes (Nutrifit, Vycon, Ecouen, France) were placed on both sedated and unsedated animals in sternal recumbency after lubricating the tip of the tube with lidocaine gel. The length (from 50 to 125 cm) and diameter (from 2.0 to 4.5 mm) were adjusted to the animal size. They were fixed with sutures on the nasal planum after application of a topical anesthetic cream containing lidocaine, radiographically confirmed, and protected by a plastic cone. The radiographic confirmation of NFT placement systematically included a right lateral view including cervical and thoracic areas, with an additional ventrodorsal view in equivocal cases. Images were reviewed by a resident in training or a board‐certified veterinary radiologist. Naso‐esophageal feeding tubes were exclusively placed in animals without vomiting, esophageal diseases, poor gag reflex, or altered mental status. Animals presented with vomiting were medically stabilized before NFT placement with intravenous antiemetic drugs.

Animals were fed with liquid diet adapted to the underlying pathological process and nutritional needs. Before each use, feeding tube placement was checked by aspirating air with an empty syringe, and by administering 2 mL of sterile saline, looking for cough. Animals were fed in sternal recumbency over a 5‐ to 10‐minute period. After each feeding, NFT were flushed with 5 mL of water to prevent tube obstruction. Animals discharged with NFT kept cone at all times. A demonstration of the use of NFT was systematically performed by a nurse or a veterinarian to the owners at the time of discharge emphasizing on tube placement verifications to prevent aspiration pneumonia. Moreover, an information document listing all the procedure was provided to the owner (Supplementary material S1). Oral tablets drugs were advised to be crushed into fine powder and mixed with water before being injected through the tube, whereas oral liquid drugs could be directly injected with a syringe.

### 2.3 NFT‐related complications

Complications associated with NFT flagged by the owners were classified into 2 categories according to their relative severity: “minor complications” (eg, mild clinical adverse effects such as vomiting/regurgitation, hypersalivation, sneezing/nasal discharge, coughing, and global discomfort characterized by any attempt of self‐removal of the tube or the cone, or NFT‐related complications such as tube displacement (outside airways), fixation issues, tube vomited out, and tube obstruction) and “major complications” (eg, life‐threatening adverse effect including displacement of the tube in airways and aspiration pneumonia). For each clinical adverse effect, a grading system was established comprising 3 possibilities to reflect the occurrence frequency: “never,” “less than once a day,” and “at least once a day.” Eventually, the number of complications (either minor or major) that led to additional consultation was also recorded.

### 2.4 Statistical analysis

Statistical analyses were performed using a R statistical software. Continuous data (eg, age, body weight, duration of NFT use during hospitalization, duration of NFT use at home, total duration of NFT use, and time from discharge to spontaneous return of appetite) were reported as median and range. Binary variables were reported as numbers with percentages. Ninety‐five percent confidence intervals (95% CI) were calculated for percentages when relevant.

## 3 RESULTS

### 3.1 Clinical data

A total of 123 animals were eligible for the study, including 94 cats and 29 dogs. Of the 94 cats, 4 were excluded because owners refused to answer questions by phone for emotional reasons after recent death of their animal. The study therefore included 119 animals (90 cats and 29 dogs).

Among cats, there were 46 neutered males, 43 neutered females, and 1 intact male. Among dogs, there were 9 neutered males, 4 intact males, 14 neutered females, and 2 intact females. Median age was 11 years (1 – 17) for the cats and 5 years (6 months – 15 years) for the dogs. Median body weight was 4.3 kg (2.1‐7.4) for the cats and 8.9 kg (2.2‐39) for the dogs. Cat breeds included 67 domestic shorthair, 4 Siamese, 3 Birmans, 3 Chartreux, 3 Persians, 2 Maine Coons, 2 British Shorthairs, 1 Turkish Angora, 1 British Longhair, 1 Exotic Longhair, 1 Scottish Fold, and 1 Scottish Longhair. Dog breeds included 5 mixed‐breeds, 3 Yorkshire Terriers, 3 Bichon Frisés, 2 Chihuahuas, 2 Shepherds, 2 Border Collies, and 1 each of Dachshund, Maltese, Beagle, Jack Russel Terrier, French Bulldog, English Bulldog, Golden Retriever, Cocker Spaniel, Korthals Griffon, Miniature Poodle, Czechoslovakian Wolfdog, and Fox Terrier.

Disease categories suspected to cause anorexia are listed in Table 1. Gastrointestinal (36/119, 30.3%), neoplastic (15/119, 12.6%), urogenital (15/119, 12.6%), and hepatobiliary (13/119, 10.9%) conditions were the most prevalent underlying processes causing loss of appetite. Clinical signs reported by the owners are reported in Table 2. Dogs and cats of the study mainly presented lethargy (86/119, 72.3%), vomiting (66/119, 55.5%), and weight loss (62/119, 52.1%). Physical abnormalities are listed in Table 3. The most common abnormal findings on physical examination were dehydration (73/119, 61.3%), abdominal pain (54/119, 45.4%), and heart murmur (38/119, 31.9%).

**TABLE 1:** Disease categories suspected to cause anorexia in the 119 animals.

Disease category	Total cases (%)	Total dogs (%)	Total cats (%)
Gastrointestinal	36 (30.3)	5 (17.2)	32 (35.6)
Neoplastic	15 (12.6)	3 (10.3)	11 (12.2)
Urogenital	15 (12.6)	2 (6.9)	13 (14.4)
Hepatobiliary	13 (10.9)	2 (6.9)	10 (11.1)
Pancreatic	6 (5.0)	4 (13.8)	3 (3.3)
Respiratory	6 (5.0)	3 (10.3)	3 (3.3)
Orthopedic	6 (5.0)	0 (0.0)	6 (6.7)
Other	6 (5.0)	4 (13.8)	2 (2.2)
Endocrine	5 (4.2)	1 (3.4)	4 (4.4)
Infectious	5 (4.2)	2 (6.9)	3 (3.3)
Neurologic	3 (2.5)	2 (6.9)	1 (1.1)
Cardiac	2 (1.7)	0 (0.0)	2 (2.2)
Hematologic	1 (0.8)	1 (3.4)	0 (0.0)

**TABLE 2:** Clinical signs of the 119 animals reported by the owners before presentation.

Clinical sign	Total cases (%)	Total dogs (%)	Total cats (%)
Lethargy	86 (72.3)	21 (17.6)	65 (54.6)
Vomiting	66 (55.5)	15 (12.6)	51 (42.9)
Weight loss	62 (52.1)	9 (7.6)	53 (44.5)
Signs of respiratory disease	25 (21.0)	8 (7.6)	17 (14.3)
Diarrhea	21 (17.6)	7 (5.9)	14 (11.8)
Polyuria/polydipsia	13 (10.9)	5 (4.2)	8 (6.7)
Signs of neurological disease	12 (10.1)	7 (5.9)	5 (4.2)
Signs of orthopedic disease	8 (6.7)	1 (0.8)	7 (5.9)
Constipation	7 (5.9)	0 (0.0)	7 (5.9)
Signs of urinary disease	6 (5.0)	0 (0.0)	6 (5.0)

**TABLE 3:** Physical abnormalities of the 119 animals at presentation.

Physical abnormality	Total cases (%)	Total dogs (%)	Total cats (%)
Dehydration	73 (61.3)	17 (70.8)	56 (62.2)
Abdominal pain	54 (45.4)	13 (54.2)	41 (45.6)
Heart murmur	38 (31.9)	8 (33.3)	30 (33.3)
Tachycardia	29 (24.4)	9 (37.5)	20 (22.2)
Hyperthermia	18 (15.1)	4 (16.7)	14 (15.6)
Pale mucous membranes	13 (10.9)	6 (25.0)	7 (7.8)
Hypothermia	12 (10.1)	3 (12.5)	9 (10.0)
Icterus	11 (9.2)	2 (8.3)	9 (10.0)
Bradycardia	9 (7.6)	1 (4.2)	8 (8.9)

### 3.2 NFT general information

Naso‐esophageal feeding tubes were kept in place while hospitalized for a median of 2 days (1‐11), while at home for a median of 6 days (1‐17), and in total for a median 8 days (2‐22). Further details on individual placement durations can be found in Supplementary material S2. Seventy‐four (62.2%, 95% CI: 53.3‐70.7) dogs and cats recovered a spontaneous appetite while having NFT at home, in a median of 4 days (1‐13) after NFT placement. Among the 45 animals who did not recover a spontaneous appetite while wearing NFT, 27 (60.0%; 95% CI: 44.4‐75.6) recovered spontaneous appetite within 24 hours after NFT removal. The remaining 18 animals that never recovered a spontaneous appetite were either hospitalized (n = 12) or euthanized (n = 6) because of worsening clinical signs (15.1% of the 119 animals) but none of them were diagnosed with aspiration pneumonia.

### 3.3 Complications

**FIGURE 1:**
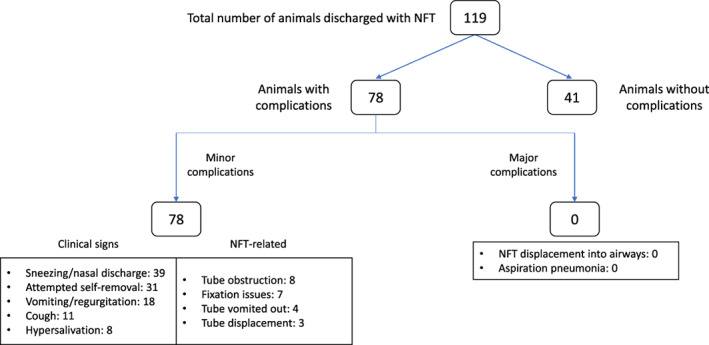
Flow diagram of the overall complication rates of naso‐esophageal feeding tubes (NFT) placement in the 119 animals discharged with NFT at home.

Overall complication rate related to NFT was 65.5% (95% CI: 57.0‐74.0) affecting 78 dogs and cats. The details for each category are summarized in Table 4 and Figure 1. Only minor complications occurred in these 78 animals, with sneezing/nasal discharge as the most prevalent. They are summarized in Table 5. Attempt to self‐removal of the NFT was reported in 31/119 animals (26.1%), all wearing a cone, but 59 owners admitted to having removed the cone under their supervision and only 10 of these 59 animals (16.9%) manifested discomfort and attempted self‐removal. Additional consultations were required for 22 animals (18.5%; 95% CI: 11.5‐25.5), but only 7 animals needed a new NFT placement because of tube displacement (outside airways) or vomited out before the return of a spontaneous appetite. No major complication occurred as no owner reported clinical signs compatible with aspiration pneumonia (eg, fever, lethargy, persistent coughing, and dyspnea) and no NFT displacement into airways was detected during the additional consultations.

**TABLE 4:** Complication rates based on the severity categories in the 119 animals after discharge.

Complication categories	Number of animals (%; 95% CI)
Minor complications	78 (65.5; 54.0‐74.0)
Clinical signs	
Sneezing/nasal discharge	39 (32.8)
Attempted self‐removal of NFT	31 (26.1)
Vomiting/regurgitation	18 (15.1)
Cough	11 (9.2)
Hypersalivation	8 (6.8)
NFT‐related	
Tube obstruction	8 (6.7)
Fixation issues	7 (5.9)
Tube vomited out	4 (3.3)
Tube dislodgement (except airways)	3 (2.5)
Major complications	0
Displacement into airways	0
Aspiration pneumonia	0

Abbreviations: CI, confidence interval; NFT, naso‐esophageal feeding tubes.

**TABLE 5:** Frequency of minor clinical complications after discharge reported by owners of the 119 animals.

Side effect	Never (%)	Less than once a day (%)	At least once a day (%)
Vomiting/regurgitation	101 (84.9)	15 (12.6)	3 (2.5)
Hypersalivation	111 (93.2)	4 (3.4)	4 (3.4)
Cough	108 (90.8)	10 (8.4)	1 (0.8)
Sneezing	80 (67.2)	27 (22.7)	12 (10.1)
Attempted self‐removal of NFT	88 (73.9)	22 (18.5)	9 (7.6)

Abbreviation: NFT, naso‐esophageal feeding tubes.

### 3.4 Owners‐related data

Ninety‐nine owners (83.2%, 95% CI: 76.5‐89.9) were the only person who managed NFT at home. Tube management was considered “simple” by 80.7% of owners (96/119), “simple after some practice” by 16.8% (20/119), and “complex” by 2.5% (3/119). One hundred fifteen owners (96.6%, 95% CI: 93.3‐99.3) considered that explanations at discharge were adequate. Mechanical difficulties for drug administration through NFT were encountered by 21 owners (20.6%) among the 102 (85.7%) that used NFT to give medications. Eventually, 94.1% (95% CI: 89.9‐98.3) of owners were globally satisfied with the use of NFT at home.

## 4 DISCUSSION

Our retrospective study describes the use of naso‐esophageal feeding tubes at home in dogs and cats. Few studies report the use of such tubes in both species,^15^, ^16^, ^17^, ^18^, ^19^ and only 1 study reports limited data in cats with suspected acute pancreatitis discharged with NFT in place.^18^ Our study provides evidence that NFT can be well tolerated at home without life‐threatening complications.

Naso‐esophageal and nasogastric tubes are often described in the veterinarian literature as only adequate for short‐term use (<5 days).^4^ However, in our study, the median total duration of NFT placement was 8 days, and some animals tolerated the tubes up to 22 days. Moreover, some other studies evaluating the use of NFT report duration times longer than current recommendations, up to 17 days in cats^15^ and 18 days in dogs.^16^ Spontaneous return of appetite while wearing NFT at home was observed in 62.2% of cases, showing that a large majority of animals were able to eat despite the presence of feeding tubes, and similar ranges (50%‐60.8%) are reported in the literature.^15^, ^17^ However, NFT seemed to generate stress or discomfort in several animals, as 60% of dogs and cats who remained anorexic while wearing NFT recovered spontaneous appetite after tube removal. However, discriminating implication of the underlying disease and NFT discomfort as the cause of anorexia can be challenging for owners and veterinarians, and the decision to remove NFT in anorexic animals should be a case‐by‐case decision. Wearing a cone can cause some discomfort that can hardly be differentiated from NFT adverse effects. Our results nevertheless revealed that only 16.9% of animals attempted self‐removal of the tubes while not wearing a cone. This information seems to suggest that NFT are only mildly responsible for the general discomfort reported.

Complications associated with NFT occurred in 65.5% of cases in our study with sneezing as the most frequent 1 (32.8%). These rates were either similar or lower than those reported by other studies.^15^, ^16^, ^17^, ^18^, ^19^ Therefore, complications related to NFT does not seem to occur more frequently at home than under the supervision of medical staff during hospitalization. Although the overall complication rate of 65.5% seems quite high, all of these adverse effects were considered minor complications with minimal impact on the quality of life and no life‐threatening adverse effects were reported. Furthermore, this rate might have been overestimated if clinical signs related to the underlying disease were counted as NFT complications. For example, cough was reported by owners in a dog treated for a cervical bite wound with tracheal injury and in a second 1 with infectious bronchopneumonia. Tube dislodgement is one of the most worrisome complications associated with NFT, especially for animals without medical supervision. In 1 study describing cats discharged with NFT,^18^ the only relevant complication was reported in a cat who self‐removed the NFT and required endoscopy to remove a segment of the tube from the stomach. Such complications were not reported in our study therefore identifying NFT dislodgement as an unusual complication, provided that NFT placement and management follow rigorous protocol along with appropriate owner education.

Aspiration pneumonia is another threatening complication associated with enteral feeding tubes. In humans, relations between the presence of a nasogastric feeding tube and aspiration pneumonia is well recognized, with several mechanisms suspected: the presence of the tube through the sphincters could cause a loss of integrity of their function, the chronic stimulation of the pharynx could predispose to reflux events and decreases protective pharyngoglottal reflexes, pharyngeal secretions could be increased, and gastric bacteria could migrate upward along the tube, colonize the pharynx, and enter the lower respiratory tract.^14^ However, no evidence of this association exists in small animal practice, and only 1 study reports 1 case of aspiration pneumonia as a complication of nasogastric tubes placement in dogs.^16^ Moreover, in a study describing the etiology and clinical outcome in dogs with aspiration pneumonia, the use of enteral feeding tubes was not reported among the 88 reported cases.^20^ The absence of aspiration pneumonia reported in our study corroborate these observations. However, as aspiration pneumonia does not necessarily require antimicrobial therapy,^21^ this specific form of pneumopathy therefore could not have been formally excluded in the 11 dogs presenting cough. It remains nevertheless unlikely as the cough was reported every day in only 1 dog. Moreover, undiagnosed aspiration pneumonia in the coughing dogs, if any, was not life‐threatening as none of the animals euthanized or required hospitalization were diagnosed with this condition.

Stringent criteria should be applied to decide whether an animal is a good candidate for NFT placement. This includes animals without severe nasal or esophageal disease, facial trauma, uncontrolled vomiting, coagulopathy, or poor gag reflex.^4^, ^5^ Tube placement must be systematically confirmed with thoracic radiographs to avoid accidental tracheal catheterization. Complications with NFT misplacement leading to iatrogenic pneumothorax have been published in dogs,^22^ including in intubated ones,^23^ but not in cats to the authors' knowledge. Even if only very few cases are reported, these observations highlight the importance of radiographic controls after tube placement and systematic verification before feeding. Moreover, NFT tolerability (eg, occurrence of clinical signs related to the presence of a tube) should be firstly monitored during hospitalization to minimize the risks of complications at home. In our study, a minimum observational period of 24 hours was part of the inclusion criteria. Eventually, discussion with owners is essential to determine if they feel comfortable with NFT management. A study conducted on cats with esophagostomy and percutaneous endoscopic gastrotomy tubes revealed that almost all owners were satisfied and comfortable with management of this kind of tubes.^24^ With adequate explanations and precise demonstration for owners, we believe that NFT are also suitable for home care. The results of our study showed that only 2.5% of owners considered the use of NFT complex and 83.2% handled the device alone, which provides strong feasibility arguments for owners. This study revealed that owners were highly satisfied with the use of NFT at home with a global satisfaction rate of 94.1%. Only 4 owners refused to answer our questions by phone, for emotional reasons. However, the low refusal participation rate of 3.3% has only minimal impact on the global satisfaction rate. For the 5.9% of unsatisfied owners, difficulties to handle the device and stress induced to their animals were quoted as reasons for unsatisfaction.

The main limitation of our study was the retrospective assessment of complications by telephone calls to owners. Some owners could have had some difficulties remembering events that occurred several months ago, leading to a potential misclassification of adverse effect occurrence. The arbitrary limit of 5 months after discharge has been established for this reason. Moreover, the 3 subcategories reflecting the frequency of occurrence of these clinical signs were large enough to encompass different ranges of adverse effect occurrence. Therefore, the possible bias associated with this retrospective assessment was considered low. Another limitation of our study was the subjective allocation of complications into 2 groups according to their relative severity. Even though some “minor” complications have led to additional consultation (eg, NFT replacement) and could be considered as major complications by owners, especially for financial reasons, the authors wanted to put emphasis on the life‐threatening complications that would adversely affect managing NFT at home.

## 5 CONCLUSION

The study provides evidence that NFT can be well tolerated at home in both dogs and cats. Only minor complications were reported, mainly sneezing, and no aspiration pneumonia occurred. Moreover, owners were highly satisfied and could easily handle the device. The use of NFT at home might represent an interesting alternative way to reduce the costs for the owners and facilitate enteral feeding and drug administration until recovery of a spontaneous appetite. However, careful case selection and confirmation of tube placement with radiography are essential to prevent complications, along with attentive owner education and support. Our results open the field to further prospective studies to compare tolerability and complications of naso‐esophageal with other feeding tubes at home.

## CONFLICT OF INTEREST DECLARATION

Authors declare no conflict of interest.

## OFF‐LABEL ANTIMICROBIAL DECLARATION

Authors declare no off‐label use of antimicrobials.

## INSTITUTIONAL ANIMAL CARE AND USE COMMITTEE (IACUC) OR OTHER APPROVAL DECLARATION

Authors declare no IACUC or other approval was needed.

## HUMAN ETHICS APPROVAL DECLARATION

Authors declare human ethics approval was not needed for this study.

## Supporting information


**Data S1:** Supporting Information

**Data S2:** Supporting Information
