# Supplementary material for: Tolerability of naso‐esophageal feeding tubes in dogs and cats at home: Retrospective review of 119 cases
Source: J Vet Intern Med. 2023 Oct 25;37(6):2315–21. doi: 10.1111/jvim.16732 (PMC10658475; doi:10.1111/jvim.16732)
Supplement: Supplementary file 1 — Data S1: Supporting Information [file JVIM-37-2315-s001.pdf]

Supplementary Material S2: Owners information document

Owners information document

The naso-esophageal feeding tube

This feeding tube is placed in one of your pet's nostrils and reaches the end of the esophagus to achieve an early enteral nutrition and to deliver nutrients until the return of a spontaneous appetite. This type of feeding tube can be placed without general anesthesia and does not require special cares once in place. A radiographic confirmation of the tube placement is systematically performed with a right lateral view of the thorax. The tube is then sutured on the nasal planum and protected with a collar. Your animal must keep the protection cone at all times while having the tube. Once your pet has recovered a spontaneous appetite, the tube can be easily removed by your referring veterinarian or in our center.
The main disadvantages of this type of refeeding tube compared to the others are the risk of obstruction due to its small diameter, the nasal discomfort, and the need to wear a collar.
Minor complications (occasional): sneezing, serous nasal discharge, self-removal of the tube, obstruction.
Major complications (rare): tubes vomited out, aspiration pneumonia after tube dislodgement.

How to feed my pet with the naso-esophageal feeding tube?

Your pet has been fed in hospital with a specific liquid diet and the same diet must be used at home.

For each use of the tube, please follow these instructions:
1/ Your animal must be kept on ventral recumbency throughout the duration of the meal. First, check that the position of the probe has not changed on your animal and that it behaves normally.
2/ Then, aspirate into the tube using an empty syringe. You should feel a "vacuum" and the piston plunger should retract by itself after release. If you can aspirate air repeatedly, contact a veterinarian.
3/ If phase 2 went well, inject 2 to 5 mL of water: your pet should not react and the fluid must flow without pressure. In the opposite situation, contact a veterinarian.
4/ The meal must be slowly administrated over a 5- to 10-minute period, making breaks if necessary.
5/ After each feeding, the tube must be flushed with water to prevent obstructions (see below).
In cats, the volume administered per meal should not exceed 60 mL.

What's the refeeding plan for my pet?

- Total daily volume of liquid food: …….. ml
  Number of meals per day: ……..
  Water volume to flush the tube: ……… ml

My pet is nauseous/vomits right after feeding: what should I do?

The volume administered or the feeding speed are probably inappropriate. In this situation, slow down the liquid diet administration et give smaller volumes for the next 24 hours. Contact a veterinarian if no improvement occurs.

The tube of my pet is obstructed: what should I do?

1/ Check that the outer part of the tube is not twisted.
2/ Check that the syringe itself is not clogged.
3/ Check that some food is not stuck in the visible part of the tube, from the stopper to the entrance to the nostril. If so, gently massage the outer part of the tube to soften the elements.
4/ If the obstruction seems deeper, apply a slightly more intense pressure using an air-filled syringe.
5/ Contact a veterinarian if the tube is still obstructed

When should I contact a veterinarian?

Contact your veterinarian or any emergency service if you notice:
- Any tube dislodgement or suture removal
- Abundant or purulent nasal discharge
- Any discomfort during feeding
- Vomiting, regurgitation, lethargy, fever
- Any obstruction, even if removed, to keep your veterinarian informed.
