# Supplementary material for: Tolerability of naso‐esophageal feeding tubes in dogs and cats at home: Retrospective review of 119 cases
Source: J Vet Intern Med. 2023 Oct 25;37(6):2315–21. doi: 10.1111/jvim.16732 (PMC10658475; doi:10.1111/jvim.16732)
Supplement: Supplementary file 2 — Data S2: Supporting Information [file JVIM-37-2315-s002.pdf]

Supplementary Material S2: Duration of NFT placement for each animal whilst in hospital, at home, and in total.

Individuals	NFT kept in place while hospitalized (days)	NFT kept in place while at home (days)	Total duration (days)
**Cat 1**	1	4	5
**Cat 2**	2	7	9
**Cat 3**	3	8	11
**Cat 4**	3	4	7
**Cat 5**	2	7	9
**Cat 6**	2	6	8
**Cat 7**	7	15	22
**Dog 1**	4	2	6
**Dog 2**	2	17	19
**Cat 8**	3	7	10
**Dog 3**	3	3	6
**Dog 4**	3	10	13
**Dog 5**	2	5	7
**Cat 9**	9	2	11
**Dog 6**	5	5	10
**Cat 10**	2	15	17
**Cat 11**	3	17	20
**Cat 12**	2	2	4
**Dog 7**	1	5	6
**Dog 8**	4	15	19
**Cat 13**	3	6	9
**Cat 14**	2	5	7
**Cat 15**	1	3	4
**Cat 16**	2	7	9
**Cat 17**	2	10	12
**Cat 18**	2	8	10
**Dog 9**	1	6	7
**Cat 19**	3	15	18
**Dog 10**	3	10	13
**Cat 20**	1	7	8
**Cat 21**	2	4	6
**Dog 11**	3	4	7
**Cat 22**	1	4	5
**Cat 23**	1	6	7
**Cat 24**	1	7	8
**Cat 25**	3	5	8
**Cat 26**	2	15	17
**Cat 27**	2	15	17
**Dog 12**	2	4	6
**Cat 28**	3	5	8
**Cat 29**	3	3	6
**Cat 30**	2	15	17
**Cat 31**	3	5	8

**Cat 32**	5	7	12
**Cat 33**	2	4	6
**Cat 34**	2	15	17
**Dog 13**	1	5	6
**Cat 35**	1	10	11
**Cat 36**	2	3	5
**Dog 14**	4	7	11
**Cat 37**	2	10	12
**Cat 38**	1	5	6
**Dog 15**	2	6	8
**Cat 39**	1	5	6
**Cat 40**	1	7	8
**Cat 41**	2	2	4
**Cat 42**	1	6	7
**Cat 43**	2	10	12
**Cat 44**	1	5	6
**Cat 45**	2	4	6
**Dog 16**	2	6	8
**Cat 46**	2	10	12
**Cat 47**	3	7	10
**Cat 48**	1	5	6
**Cat 49**	1	8	9
**Dog 17**	2	5	7
**Cat 50**	2	8	10
**Dog 18**	3	6	9
**Cat 51**	2	7	9
**Cat 52**	2	4	6
**Dog 19**	2	7	9
**Cat 53**	3	5	8
**Cat 54**	2	9	11
**Dog 20**	1	1	2
**Cat 55**	3	2	5
**Cat 56**	1	5	6
**Dog 21**	3	10	13
**Cat 57**	2	3	5
**Cat 58**	2	7	9
**Cat 59**	2	7	9
**Cat 60**	5	5	10
**Cat 61**	1	15	16
**Cat 62**	2	8	10
**Cat 63**	2	15	17
**Cat 64**	3	2	5
**Cat 65**	1	15	16
**Cat 66**	1	4	5
**Cat 67**	2	6	8
**Cat 68**	7	2	9
**Cat 69**	1	4	5
**Cat 70**	2	5	7
**Cat 71**	5	8	13
**Cat 72**	3	5	8

**Dog 22**	1	7	8
**Dog 23**	11	4	15
**Dog 24**	2	6	8
**Cat 73**	2	5	7
**Cat 74**	2	7	9
**Cat 75**	2	7	9
**Cat 76**	2	2	4
**Cat 77**	3	5	8
**Cat 78**	1	7	8
**Cat 79**	5	5	10
**Cat 80**	1	5	6
**Cat 81**	2	15	17
**Cat 82**	3	10	13
**Cat 83**	6	8	14
**Cat 84**	2	7	9
**Cat 85**	4	2	6
**Dog 25**	2	2	4
**Cat 86**	2	5	7
**Cat 87**	3	7	10
**Dog 26**	3	4	7
**Dog 27**	3	8	11
**Cat 88**	2	8	10
**Cat 89**	2	4	6
**Dog 28**	1	3	4
**Dog 29**	3	2	5
**Cat 90**	2	15	17
